# Neurological outcomes analysis of HTLV-1 seropositive patients of the Interdisciplinary Research HTLV Group (GIPH) cohort, Brazil

**DOI:** 10.1186/1742-4690-11-S1-P51

**Published:** 2014-01-07

**Authors:** Débora B Reiss, Gabriela S Freitas, Rafael HC Bastos, Mariana A de Souza, Cláudia LF Horiguchi, Marina L Martins, Anísia SD Ferreira, Ana Lúcia B Starling, Luiz Cláudio F Romanelli, Anna Bárbara FC Proietti

**Affiliations:** 1 Faculdade da Saúde e Ecologia Humana (FASEH), Vespasiano, Minas Gerais, Brazil; 2 Grupo Interdisciplinar de Pesquisa em HTLV (GIPH), Belo Horizonte, Minas Gerais, Brazil

## 

HTLV-1 Associated Myelopathy/Tropical spastic paraparesis (HAM/TSP) is the classical neurological syndrome associated with this retrovirus, although other neurological complications are described. We collected data in the neurological and epidemiological databases related of the GIPH cohort, from 1997 and 2012, as well as in the records of the participants. Statistics involved a 95% confidence interval (CI). The main neurological outcomes presented by seropositive patients in the cohort were described and a comparison between groups of patients with and without HAM/TSP was performed with regard to other clinical manifestations. Among the 255 cohort participants examined by a neurologist, 233 (91%) were positive for HTLV-1 (CI 88-95%). From those, 12% had HAM/TSP (CI 8-16%), 20% had low back pain (CI 15-25%); 18% were diagnosed with depression, cramps and fatigue (CI 13-23%); 14% had myalgia (CI 10-18%); 13% had acute pain (CI 9-17%), 11% had urinary incontinence (CI 7-15%) and 10% had hearing loss (CI 6-14%). Comparing groups of patients with and without HAM/TSP, we found that symptomatic myelopathy patients had a greater chance of developing events such as urinary retention (RR= 9.8), gait abnormalities (RR= 7.3), constipation (RR= 6.7), urinary incontinence (RR= 5.0), sexual dysfunction (RR= 3.2), fatigue (RR= 2.9) and myalgia (RR= 2.7), when compared with asymptomatic to HAM/TSP (p<0.0001). In conclusion, 12% of patients developed HAM/TSP, which is a higher rate than that described in the literature. Furthermore, we found that patients with HAM/TSP had higher risk to develop other neurological outcomes related to this virus infection.

